# Racial and ethnic inequities of palliative care use among advanced Non‐Small cell lung cancer patients in the US

**DOI:** 10.1002/cam4.5538

**Published:** 2022-12-19

**Authors:** Jessica Y. Islam, Dejana Braithwaite, Dongyu Zhang, Yi Guo, Tina D. Tailor, Tomi Akinyemiju

**Affiliations:** ^1^ Center for Immunization and Infections in Cancer, Cancer Epidemiology Program, H. Lee Moffitt Cancer Center Tampa Florida USA; ^2^ Department of Population Health Sciences Duke University School of Medicine Durham North Carolina USA; ^3^ Department of Epidemiology, College of Public Health and Health Professions and College of Medicine University of Florida Gainesville Florida USA; ^4^ Department of Health Outcomes and Biomedical Informatics, College of Medicine University of Florida Gainesville Florida USA; ^5^ Department of Radiology Duke University School of Medicine Durham North Carolina USA

**Keywords:** equitable cancer care, palliative care, racial/ethnic inequities, supportive cancer care

## Abstract

**Background:**

With early intervention, palliative care (PC) can improve quality of life and increase survival among advanced‐stage non‐small cell lung cancer (aNCSLC) patients. However, PC is often offered late in the cancer treatment course and is underused. We characterized racial/ethnic inequities and the role of healthcare access in PC use among patients with aNSCLC.

**Methods:**

We used data from the 2004–2016 National Cancer Database, including adults aged 18–90 years with aNSCLC (stage 3 or 4 at diagnosis; *n* = 803,618). Based on the NCCN guidelines, PC includes non‐curative surgery, radiation, chemotherapy, pain management, or any combination of non‐curative care. We examined PC use by sociodemographic and health care‐level characteristics. To evaluate the independent associations of race/ethnicity and health care access characteristics with PC, we estimated adjusted odds ratios (aOR) with 95% confidence intervals (95% CI). Covariate adjustment sets varied by exposure determined using directed acyclic graphs.

**Results:**

Our population was 55% male and 77% non‐Hispanic/Latinx (NH)‐White, with a mean age of 68 years. Overall, 19% of patients with aNSCLC used PC. Compared to NH‐White patients, NH‐Black (aOR:0.91,95% CI:0.89–0.93) and Hispanic/Latinx (aOR:0.80,95% CI:0.77–0.83) patients were less likely to use PC, whereas Indigenous (AI/AN) (aOR:1.18,95% CI:1.06–1.31) and Native Hawaiian/Pacific Islander (aOR:2.08,95% CI:1.83–2.36) patients were more likely. Overall, compared to the privately‐insured, uninsured (aOR:1.19,95% CI:1.11–1.28) and Medicaid‐insured patients (aOR:1.19,95% CI:1.14–1.25) were more likely to use PC.

**Conclusion:**

PC is underutilized among NH‐Black and Hispanic/Latinx patients with aNSCLC. Insurance type may play a role in PC use among patients with aNSCLC.

## Graphical abstract

Racial/ethnic inequities exist in palliative care utilization among patients with advanced‐stage non‐small cell lung cancer, US National Cancer Database (2004–2016).

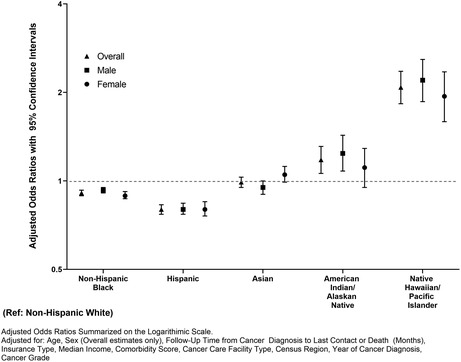

## 1 BACKGROUND

In the United States (US), lung cancer is the leading cause of cancer‐related death in men and women,^1^ often due to late‐stage disease at detection. When diagnosed at advanced‐stage, non‐small cell lung cancer (aNSCLC) is a debilitating disease that results in a high burden of symptoms and poor survival; five‐year survival after diagnosis of distant NSCLC tumors is only 5%.^2^ patients with aNSCLC frequently experience a high symptom burden during their treatment course, and these symptoms differ across illness trajectories and treatment regimens.^3^ For example, following a thoracotomy, pain is reported among 90% of NSCLC patients.^4^, ^5^ Other symptoms occurring after surgical interventions may include aching and burning sensations or neuropathic symptoms, reported in 40–69% of patients.^6^, ^7^ NSCLC patients also frequently experience cough, dyspnea, wheeze, and hemoptysis, reported in 40 to 85% of patients.^8^ High patient reported symptom burden greatly affects quality‐of‐life and is associated with shorter survival among patients with aNSCLC, underscoring the significance of addressing these outcomes.^9^, ^10^

Palliative care can alleviate symptom burden, improve patient quality‐of‐life, increase overall length of survival, and can also address care satisfaction from the perspective of both the patient and their caregiver.^11^, ^12^, ^13^, ^14^, ^15^, ^16^, ^17^ The National Comprehensive Cancer Network (NCCN) recommends intervention with palliative care should begin at cancer diagnosis to provide enhanced patient‐centered care and improve aNSCLC outcomes.^18^ However, there are significant barriers to timely receipt of palliative care among patients with aNSCLC. The most significant barrier is misconception of palliative treatments among both patients and oncologists; accepting palliative care is frequently conflated with poor prognosis.^19^ Due to these barriers racial inequities in palliative care receipt exist in the US; however, existing research has largely focused on palliative care in the context of hospice care or care provided during the end‐of‐life.^20^ Although evidence regarding patterns of palliative care use early in the care continuum specifically is limited, prior studies show that patients from minoritized communities are least likely to both discuss and use hospice care compared to others.^19^

Little is known regarding the role of health care‐level access factors in the context of palliative intervention use among patients with aNSCLC, specifically when elucidating racial and ethnic inequities. Evaluating patient‐level health care access in the context of palliative care is important, as identifying potential health care‐level determinants may inform targeted intervention development to improve access to equitable palliative care. Further, it is important to contextualize the societal structures from a health care perspective that influence racial inequities in palliative care use. Differences in cancer care quality by racial/ethnic groups do not have a biological etiology, but rather are manifestations of structural and societal barriers minoritized communities face in the U.S.^21^ Our objective was to characterize racial and ethnic inequities in palliative care use among patients with advanced NSCLC. We further estimated odds of receiving curative treatment among those patients who received and did not receive palliative care. We characterized overall and race/ethnicity stratified associations of health care access with palliative care utilization. We considered the following measures of health care access, patient's type of insurance, distance from patient to provider, type of cancer treatment facility, and Medicaid expansion status of the state where treatment occurred. We hypothesized indicators of low health care access, for example being uninsured or living a further distance from their provider, would be associated with lower palliative care use. Insights gained from this research will support the future development of health care system or provider‐level interventions to optimize equitable palliative cancer care services.

## 2 METHODS

### 2.1 Data source

To conduct our analysis, we leveraged the 2004–2016 National Cancer Data Base (NCDB) Participant Use Files (PUF). The NCDB is a US clinical oncology database collecting electronic health record patient‐level data of cancer patients treated at one of over 1500 Commission on Cancer (CoC) accredited institutions.^22^, ^23^ Achieving CoC accreditation indicates that the hospital is able to provide a multidisciplinary team approach care, long‐term patient monitoring and follow‐up, access to the latest advancements to cancer treatment, availability of psychosocial support and survivorship for patients, and prioritizes continuous quality improvements in care. The NCDB databases captures over 29 million independent cancer cases, which is estimated to include about 72% of all patients with newly diagnosed cancer.^22^ The data included in the NCDB registry are standardized and collected from patient electronic health records. Certified tumor registrars (CTR), trained in cancer registry operations, conduct data abstraction from patient's electronic health records using standardized methods to be included in the NCDB.^24^ The study was approved by the Institutional Review Board at Duke University under a general protocol (IRB#: Pro00102834).

### 2.2 Study population

We included patients with advanced (primary stage III and IV at diagnosis) non‐small cell lung cancer (aNSCLC) at cancer diagnosis. Patients diagnosed between January 1, 2004, to December 31, 2016 with NSCLC based on the International Classification of Diseases for Oncology, Third Edition topography codes C340, C341, C342, C343, C348, and C349 were included. We excluded patients with missing or unknown cancer stage (*n* = 99,804, 6.5%). Additionally, patients with missing data on palliative care utilization (*n* = 3380, 0.4%) were also excluded.

### 2.3 Study variables

Our primary outcome was palliative treatment, which is characterized by the NCDB as treatments with noncurative intent based on whether the patient's medical records explicitly mentioned that the goal of the provided treatment is palliation and not cure. Specifically, treatment is categorized as palliative if the treatment was provided to “prolong a patient's life by controlling symptoms, to alleviate pain, or to make the patient more comfortable.^25^” The NCDB ensures that types of palliative care include noncurative pain management, surgery, radiation, or systemic treatment administered to alleviate symptoms. Patients who receive palliative care may also contemporaneously undergo curative cancer treatment. The NCDB includes information on any palliative care from patients' clinical medical records during their treatment at the reporting facility. Data included in the NCDB do not capture hospice services or referral and were not included in the definition of palliative care.

Receipt of first‐line curative cancer treatment was defined as surgery, radiotherapy, systemic therapy, or any combination of these therapies. For the present analysis, several health care access variables were evaluated as described here. Type of insurance was defined as the patient's primary payor at the time of first treatment and/or cancer diagnosis. Distance from patient to provider was defined as greatest circle distance or the distance (miles) between the patient's residence (based on the latitude or longitude of the centroid of either the patient's zip code centroid or city if the zip code was not available) and the provider's hospital location (based on hospital street address). Type of cancer treatment facility included community cancer programs, comprehensive community cancer programs, academic or research programs, or integrated network cancer programs. The NCDB includes a measure capturing Medicaid expansion status of state the patient and categorizes states as non‐expansion states, early expansion states (2010–2013), January 2014 expanded states, and late expansion states (after January 2014). As the Affordable Care Act was enacted in 2011, we included data starting from January 1st, 2011 to December 31st 2016 when modeling Medicaid expansion as our main exposure.^26^ We have included the study characteristics of this sub‐sample in Supplementary Table 1.

We defined race and ethnicity using the following categories based on data availability of the NCDB: Non‐Hispanic/Latinx White (NH‐White), NH‐Black, Hispanic/Latinx, Asian, American Indian/Alaskan Native, Native Hawaiian/Pacific Islander, and other Race. When evaluating individual health care access measures, we compared estimates across racial and ethnic groups, specifically NH‐White, NH‐Black, Hispanic/Latinx, and Asian patients with aNSCLC with cancer, to ensure adequate sample size for statistical modeling.

### 2.4 Statistical analysis

We descriptively summarized patient sociodemographics and clinical cancer characteristics as percentages stratified by receipt of palliative care. We also conducted descriptive analyses to characterize racial and ethnic differences in palliative care use using Pearson Chi‐squared tests. We evaluated racial and ethnic disparities in palliative care overall and by sex using multivariable logistic regression modeling after adjustment for age (continuous), months from cancer diagnosis at last contact or death, insurance type, median income, Charlson‐Deyo comorbidity score, cancer care facility type, census region, year of cancer diagnosis and cancer grade. We also evaluated racial ethnic inequities in first‐line curative treatment receipt among patients with aNSCLC overall, and stratified by palliative care receipt status using the same adjustment set. Next, we estimated associations of health care access measures with palliative care use using multivariable logistic regression modeling. To identify the minimal sufficient adjustment sets for each health care access measure exposure model, we used directed acyclic graphs or DAGs^27^ constructed based on prior literature describing determinants of palliative care. The final DAGs are included in the Supplementary Material. For each model, we calculated cluster‐robust standard errors to account for non‐independence within clusters at the facility level to adjust for correlated patient characteristics within hospitals. All analyses were performed with Stata statistical software, version 15.0 (StataCorp).

## 3 RESULTS

Overall, we included 899,521 patients with aNSCLC with an average age of 67.9 years (SD = 11.1) (Table 1). About 45% of patients with aNSCLC were female, and 76% were NH‐White. Eleven percent of patients with aNSCLC were NH‐Black, 3% were Hispanic/Latinx, and 2% were Asian. Overall, 19% of patients with aNSCLC used palliative care. The majority utilized either surgery, radiation, or chemotherapy alone as palliative care. Palliative care in the form of pain management was utilized by 6% of patients with aNSCLC. Hispanic/Latinx patients with aNSCLC had the lowest prevalence of palliative care utilization (16%), whereas Native Hawaiian/Pacific Islander (27%) had the highest. Patients utilizing palliative care most commonly were uninsured (22%) or Medicaid‐insured (22%). We observed an increasing trend of palliative care use over time from 2004 (16%) to 2016 (21%). Six percent of patients with Stage III aNSCLC utilized palliative care, whereas 26% of Stage IV patients utilized palliative care.

**TABLE 1:** Characteristics among patients with advanced stage (Stage 3/4 or metastatic) non‐small cell Lung Cancer with known palliative care receipt status (*n* = 899,521)

	No palliative care (*n* = 728,498)		Received palliative care (*n* = 171,023)		Total (*n* = 899, 521)	
	No.	Row %	No.	Row %	No.	Col %
Age (Mean, SD)	67.8, 11.1		66.9, 11.3		67.9, 11.1	
Palliative care provided (Col %)						
No palliative care	728,498	100.0	0	0.0	728,498	81.0
Surgery/radiation/chemo only	0	0.0	129,209	75.6	129,209	14.4
Pain management only	0	0.0	10,208	6.0	10,208	1.1
Combination of surg/rad/chemo and pain management	0	0.0	21,106	12.3	21,106	2.3
Type unknown	0	0.0	10,500	6.1	10,500	1.2
Received Curative Treatment						
Yes, received surgery/radiation/or systemic therapy	538,162	78.7	146,045	21.4	684,207	76.1
No	190,186	88.4	24,975	11.6	215,161	23.9
Sex						
Male	399,932	80.7	95,600	19.3	495,532	55.1
Female	328,566	81.3	75,423	18.7	403,989	44.9
Months from Diagnosis to Last Contact or Death						
>6 months	283,905	75.7	91,381	24.3	375,286	41.7
6–24 months	252,425	83.0	51,638	17.0	304,063	33.8
>24 months	192,168	87.3	28,004	12.7	220,172	24.5
Race and Ethnicity						
Non‐Hispanic/Latinx White	555,267	80.7	132,922	19.3	688,189	76.5
Non‐Hispanic/Latinx Black	83,078	81.2	19,215	18.8	102,293	11.4
Hispanic/Latinx	22,292	84.5	4083	15.5	26,375	2.9
Asian	17,289	83.5	3404	16.5	20,693	2.3
American Indian/Alaskan Native	1854	79.1	489	20.9	2343	0.3
Native Hawaiian/Pacific Islander	991	73.4	359	26.6	1350	0.2
Other Race	44,508	81.8	9873	18.2	54,381	6.0
Missing	3219	82.6	678	17.4	3897	0.4
Primary Health Insurance Payor						
Not Insured	27,024	78.1	7593	21.9	34,617	3.8
Private Insurance/Managed Care	203,088	80.9	47,816	19.1	250,904	27.9
Medicaid	50,180	78.0	14,173	22.0	64,353	7.2
Medicare	423,869	81.5	96,456	18.5	520,325	57.8
Other Government	11,058	80.6	2660	19.4	13,718	1.5
Insurance Status Unknown	13,279	85.1	2325	14.9	15,604	1.7
Percent Without a High School Degree Residing in Zip Code (Quartiles) 2012–2016 (Area‐Level)						
> = 17.6%	166,503	82.3	35,783	17.7	202,286	22.5
10.9–17.5%	205,842	80.9	48,556	19.1	254,398	28.3
6.3–10.8%	200,736	80.4	48,848	19.6	249,584	27.7
<6.3%	145,704	80.3	35,764	19.7	181,468	20.2
Missing	9713	82.4	2072	17.6	11,785	1.3
Median Income of Adults Residing in Zip Code (Quartiles) 2012–2016 (Area‐Level)						
< $40,227	159,956	81.0	37,535	19.0	197,491	22.0
$40,227‐50,353	172,979	80.8	41,009	19.2	213,988	23.8
$50,354‐63,332	167,326	80.7	40,064	19.3	207,390	23.1
> = $63,333	216,890	81.3	49,988	18.7	266,878	29.7
Missing	11,347	82.4	2427	17.6	13,774	1.5
Rurality						
Urban	694,991	81.0	163,016	19.0	858,007	95.4
Rural	15,618	79.5	4022	20.5	19,640	2.2
Missing	17,889	81.8	3985	18.2	21,874	2.4
State Medicaid Expansion Status (2011–2016)^a^						
Non‐Expansion States	138,147	80.1	34,397	19.9	172,544	38.7
January 2014 Expansion States	108,049	77.4	31,512	22.6	139,561	31.3
Early Expansion States (2010–2013)	56,219	84.6	10,275	15.5	66,494	14.9
Late Expansion States (after Jan. 2014)	49,477	77.1	14,688	22.9	64,165	14.4
Suppressed for Ages 0–39	2100	78.5	577	21.6	2677	0.6
Distance from Patient to Provider (Crowfly)						
<2 miles	81,461	80.8	19,388	19.2	100,849	11.2
2–4 miles	154,112	81.1	35,872	18.9	189,984	21.1
5–9 miles	158,584	81.3	36,493	18.7	195,077	21.7
10–19 miles	139,930	80.4	34,102	19.6	174,032	19.3
20–45 miles	116,932	80.4	28,553	19.6	145,485	16.2
>45 miles	77,479	82.3	16,615	17.7	94,094	10.5
Charlson‐Deyo score						
0	443,522	81.3	101,845	18.7	545,367	60.6
1	188,598	80.6	45,467	19.4	234,065	26.0
2	66,969	80.5	16,222	19.5	83,191	9.2
>=3	29,409	79.7	7489	20.3	36,898	4.1
Treatment facility type						
Community Cancer Program	83,328	82.5	17,664	17.5	100,992	11.2
Comprehensive Community Cancer Program	323,653	81.4	73,863	18.6	397,516	44.2
Academic/Research Program	218,132	80.5	52,684	19.5	270,816	30.1
Integrated Network Cancer Program	98,678	79.4	25,611	20.6	124,289	13.8
Missing	4707	79.7	1201	20.3	5908	0.7
Census region						
Northeast	144,109	77.6	41,654	22.4	185,763	20.7
South	284,996	82.2	61,691	17.8	346,687	38.5
Midwest	192,968	79.1	51,030	20.9	243,998	27.1
West	101,718	86.8	15,447	13.2	117,165	13.0
Missing	4707	79.7	1201	20.3	5908	0.7
Year of diagnosis						
2004	49,884	83.8	9672	16.2	59,556	6.6
2005	51,697	83.9	9884	16.1	61,581	6.8
2006	51,293	83.0	10,472	17.0	61,765	6.9
2007	51,203	82.1	11,180	17.9	62,383	6.9
2008	55,795	82.0	12,276	18.0	68,071	7.6
2009	57,443	81.8	12,814	18.2	70,257	7.8
2010	57,191	81.2	13,276	18.8	70,467	7.8
2011	57,495	80.8	13,636	19.2	71,131	7.9
2012	58,442	80.2	14,391	19.8	72,833	8.1
2013	59,050	79.1	15,561	20.9	74,611	8.3
2014	60,047	78.7	16,217	21.3	76,264	8.5
2015	60,682	79.0	16,125	21.0	76,807	8.5
2016	58,276	79.0	15,519	21.0	73,795	8.2
Grade						
Well differentiated, differentiated, NOS	20,861	88.0	2851	12.0	23,712	2.6
Moderately differentiated, moderately well differentiated, intermediate differentiation	99,814	85.9	16,364	14.1	116,178	12.9
Poorly differentiated	215,143	82.1	46,960	17.9	262,103	29.1
Undifferentiated, anaplastic	12,242	81.7	2746	18.3	14,988	1.7
Cell type not determined, not stated or not applicable, unknown primaries, high grade dysplasia	380,438	78.8	102,102	21.2	482,540	53.6
NCDB analytic stage group						
Stage III	300,554	93.6	20,464	6.4	321,018	35.7
Stage IV	427,944	74.0	150,559	26.0	578,503	64.3

^a^ Data were restricted to 2011–2016 as the Affordable Care Act started in 2011 (*n* = 445,441).

**FIGURE 1:**
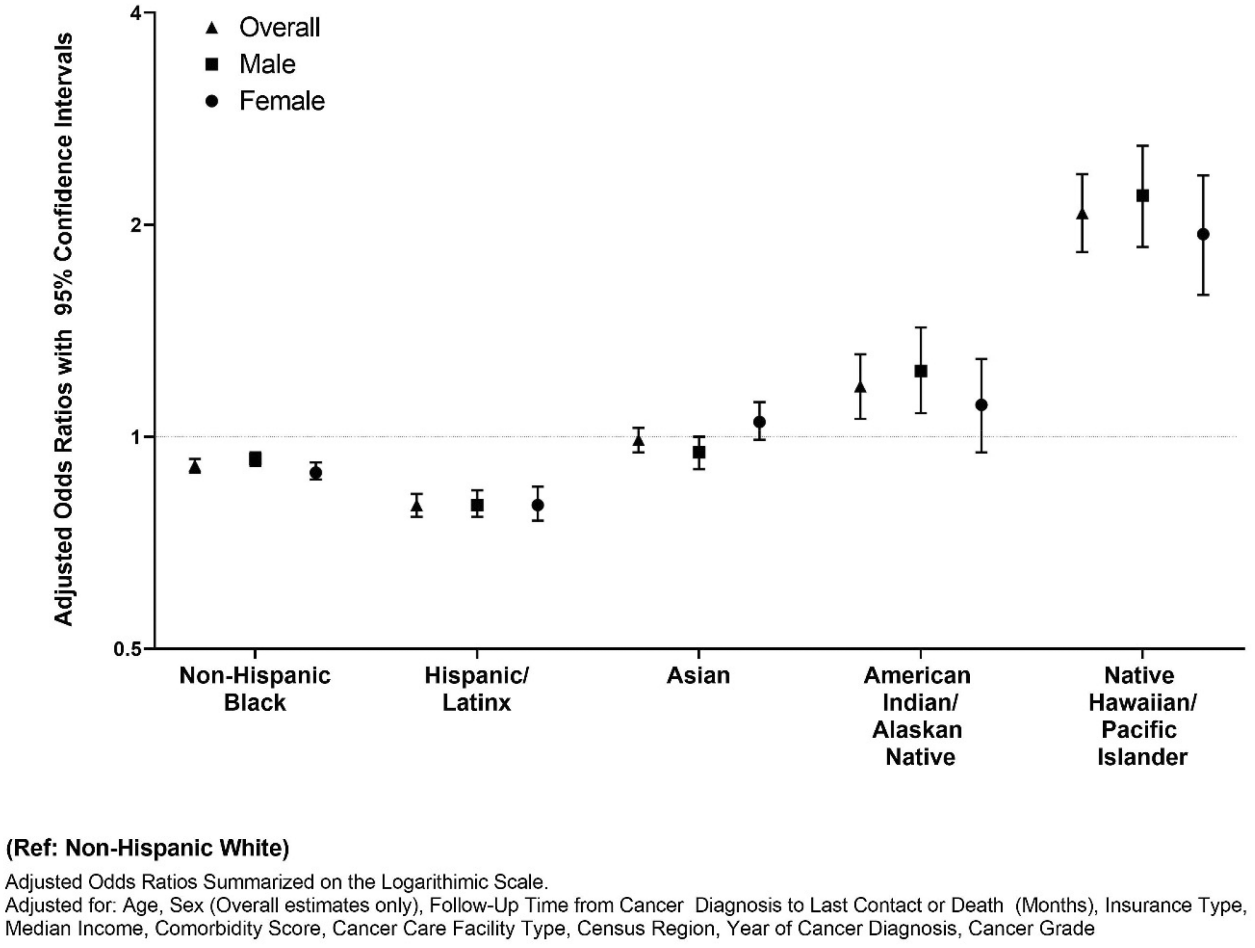
Sex‐stratified racial and ethnic inequities in palliative care utilization among patients with advanced‐stage non‐small cell lung cancer, National Cancer Database (2004–2016).

**FIGURE 2:**
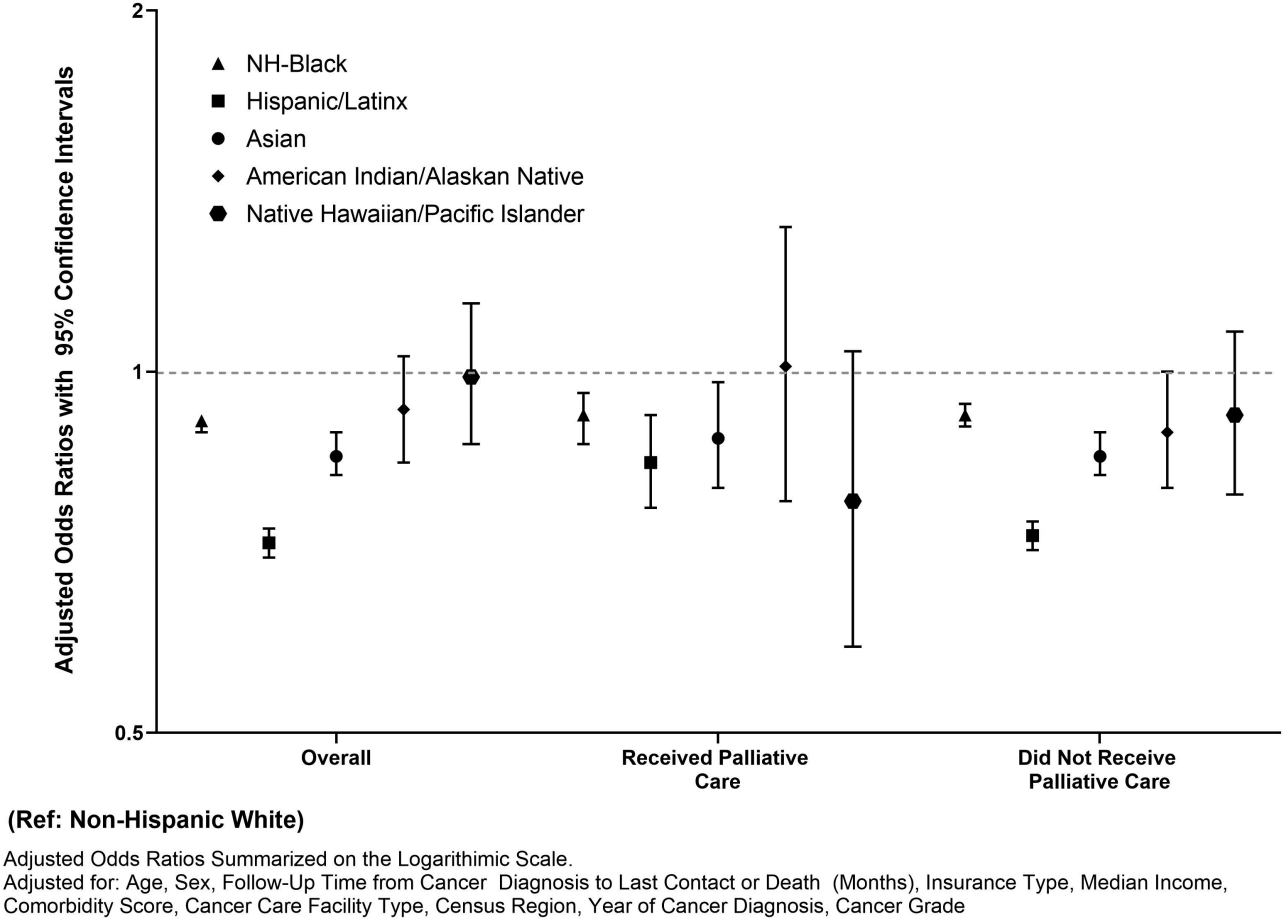
Associations of Treatment Receipt (First‐Line Surgery, Radiotherapy, or Systemic Therapy) with Race/Ethnicity Overall and by Palliative Care Receipt, National Cancer Database (2004–2016).

We estimated associations of palliative care utilization by race/ethnicity overall and stratified by sex (Figure 1). Overall, NH‐Black patients had 9% relative lower odds (aOR:0.91, 95% CI:0.89–0.93) and Hispanic/Latinx patients had 20% relative lower odds (aOR:0.80, 95% CI:0.77–0.83) of using palliative care compared to NH‐White patients. Conversely, American Indian/Alaskan Native (aOR:1.12, 95% CI:1.01–1.25) and Native Hawaiian/Pacific Islander (aOR:2.11, 95% CI:1.84–2.41) had higher odds of palliative care use compared to their NH‐White counterparts. When stratified by sex, similar trends were observed in across most racial/ethnic categories patients. When we evaluated odds of receiving first‐line curative treatment (surgery/radiotherapy/systemic therapy) among all patients with aNSCLC, we observed that compared to NH‐White patients with aNSCLC, NH‐Black (aOR:0.91, 95% CI: 0.89–0.92), Hispanic/Latinx (aOR: 0.72, 95% CI: 0.70–0.74), Asian (aOR: 0.85, 95% CI: 0.82–0.89) adults were less likely to receive first‐line curative treatment (Figure 2). These associations were consistent among those who did and did not receive palliative care.

Table 2 summarizes associations of health care utilization measures with palliative care use among patients with aNSCLC. Compared to patients with aNSCLC with private insurance or managed care, the uninsured (aOR:1.19, 95% CI:1.11–1.28) and those on Medicaid (aOR:1.19, 95% CI:1.14–1.25) were more likely to utilize palliative care, and these associations were consistent across sex. Patients, including both men and women, residing farthest (over 45 miles) from their provider were 11% less likely to use palliative care compared to those residing <2 miles from their provider (aOR:0.89, 95% CI:0.80–0.98). There were no associations between cancer treatment facility type and palliative care utilization.

**TABLE 2:** Associations of health care utilization measures with palliative care use among advanced stage non‐small cell lung cancer patients (*n* = 899,521)

	Overall		Male		Female	
	OR	95% CI	OR	95% CI	OR	95% CI
Primary payer^a^						
Not Insured	1.19	1.11–1.28	1.19	1.11–1.29	1.17	1.08–1.28
Private Insurance/Managed Care	Ref.		Ref.		Ref.	
Medicaid	1.19	1.14–1.24	1.21	1.15–1.28	1.17	1.11–1.23
Medicare	1.04	1.01–1.07	1.05	1.02–1.09	1.05	1.01–1.08
Other Government	1.04	0.94–1.14	1.03	0.93–1.15	1.01	0.90–1.13
State Medicaid Expansion Status (2010–2016)^a^ ^,^ ^b^						
Non‐Expansion States	Ref.		Ref.		Ref.	
January 2014 Expansion States	1.02	0.88–1.19	1.01	0.87–1.18	1.04	0.90–1.19
Early Expansion States (2010–2013)	0.65	0.53–0.81	0.64	0.52–0.79	0.67	0.54–0.83
Late Expansion States (after Jan. 2014)	0.99	0.84–1.17	0.99	0.83–1.17	0.99	0.85–1.18
Distance from Patient to Provider (Crowfly)^c^						
<2 miles	Ref		Ref.		Ref.	
2–4 miles	0.99	0.93–1.05	0.99	0.93–1.06	0.98	0.92–1.04
5–9 miles	0.97	0.92–1.03	0.97	0.91–1.03	0.98	0.92–1.04
10–19 miles	1.02	0.96–1.08	1.03	0.97–1.10	0.99	0.93–1.06
20–45 miles	1.01	0.94–1.08	1.02	0.93–1.10	0.99	0.92–1.07
>45 miles	0.89	0.80–0.98	0.89	0.80–0.98	0.89	0.80–0.98
Facility type^d^						
Community cancer program	0.89	0.78–1.01	0.89	0.78–1.01	0.88	0.78–0.99
Comprehensive Community Cancer Program	Ref		Ref.		Ref.	
Academic/Research Program	0.98	0.86–1.11	0.99	0.87–1.13	0.97	0.85–1.10
Integrated Network Cancer Program	1.11	0.96–1.27	1.13	0.98–1.31	1.09	0.95–1.24

^a^ Adjusted for age, race/ethnicity, and median household income quartile of patient's zip code.

^b^ Data were restricted to 2011–2016 as the Affordable Care Act started in 2011 (*n* = 445,441). Adjusted for age and census region.

^c^ Adjusted for age, race/ethnicity, area of residence (urban/rural) and census region.

^d^ Adjusted insurance type, area of residence (urban/rural), census region, and greatest circle distance to care.

Across racial/ethnic categories, patients with aNSCLC were mostly Medicare insured or insured with private insurance/managed care. NH‐Black and American Indian/Alaskan Native patients with aNSCLC mostly (>60%) lived in areas with low median household income, and over half lived in states without Medicaid expansion (Table 3). Table 4 summarizes results of each multivariable logistic model and includes the covariate adjustment set for each individual model. Compared to those with private insurance or managed care, Medicaid‐insured NH‐White (aOR:1.21, 95% CI:1.16–1.26) and NH‐Black (aOR:1.21, 95% CI:1.12–1.32) were more likely to utilize palliative care. Uninsured NH‐White (aOR:1.18, 95% CI:1.11–1.26) and NH‐Black (aOR:1.17, 95% CI:1.03–1.34) were also more likely to utilize palliative care compared to privately insured patients. Compared to patients treated at a comprehensive community cancer program, our data suggest Hispanic/Latinx (aOR:1.40, 95% CI:1.05–1.84) patients with aNSCLC treated at Integrated Network Cancer Programs were more likely to use palliative care.

**TABLE 3:** Distribution of health care access measures among advanced stage non‐small cell lung cancer patients by race/ethnicity (*n* = 841,243)

	NH‐White		NH‐Black		Hispanic/Latinx		Asian		American Indian/Alaskan Native		Native Hawaiian/Pacific Islander	
	*n*	Col %	*n*	Col %	*n*	Col %	*n*	Col %	*n*	Col %	*n*	Col %
Primary payer												
Not insured	22,066	3.2	6856	6.7	2377	9.0	1104	5.3	95	4.1	61	4.5
Private Insurance/Managed Care	192,788	28.0	25,947	25.4	6665	25.3	7027	34.0	485	20.7	466	34.5
Medicaid	38,541	5.6	15,298	15.0	3976	15.1	2683	13.0	270	11.5	203	15.0
Medicare	413,647	60.1	50,285	49.2	12,545	47.6	9323	45.1	1164	49.7	579	42.9
Other Government	10,498	1.5	1690	1.7	247	0.9	190	0.9	265	11.3	18	1.3
Insurance Status Unknown	10,649	1.5	2217	2.2	565	2.1	366	1.8	64	2.7	23	1.7
State Medicaid Expansion Status (2011–2016)^a^												
Non‐Expansion States	132,969	38.2	25,798	48.1	5635	39.2	2088	17.5	636	47.7	93	12.3
January 2014 Expansion States	111,684	32.1	14,346	26.8	3740	26.5	4136	34.6	278	20.8	422	55.8
Early Expansion States (2010–2013)	50,296	14.5	5173	9.7	3875	27.4	4943	41.4	246	18.4	201	26.6
Late Expansion States (after Jan. 2014)	51,669	14.8	7849	14.6	565	4.0	560	4.7	160	11.9	31	4.1
Suppressed for Ages 0–39	1555	0.5	461	0.9	303	2.2	228	2.2	14	1.1	9	1.2
Distance from Patient to Provider (Crowfly)												
<2 miles	71,245	10.4	16,857	16.5	3810	14.4	2839	13.7	174	7.4	95	7.0
2–4 miles	134,648	19.6	29,510	28.8	6902	26.2	6296	30.4	282	12.0	373	27.6
5–9 miles	144,922	21.1	25,295	24.7	6716	25.5	5500	26.6	265	11.3	302	22.4
10–19 miles	139,625	20.3	13,747	13.4	4720	17.9	3801	18.4	362	15.5	274	20.3
20–45 miles	120,320	17.5	10,461	10.2	2373	9.0	1352	6.5	484	20.7	148	11.0
>45 miles	77,429	11.3	6423	6.3	1854	7.0	905	4.4	776	33.1	158	11.7
Facility type												
Community Cancer Program	81,992	12.0	8445	8.3	2379	9.2	2174	10.7	437	18.8	184	13.8
Comprehensive Community Cancer Program	318,190	46.5	34,410	33.9	9623	37.3	6881	33.9	1249	53.8	418	31.4
Academic/Research Program	189,712	27.7	44,121	43.5	9957	38.6	8915	44.0	468	20.2	615	46.1
Integrated Network Cancer Program	94,677	13.8	14,460	14.3	3832	14.9	2299	11.3	168	7.2	116	8.7

^a^ Data were restricted to 2011–2016 as the Affordable Care Act was started in 2011 (*n* = 445,441).

**TABLE 4:** Associations of health care access indicators with palliative care receipt among advanced non‐small cell lung cancer patients by race/ethnicity

	NH‐White			NH‐Black			Hispanic/Latinx			Asian			American Indian/Alaskan Native			Native Hawaiian/Pacific Islander		
	OR	95% CI		OR	95% CI		OR	95% CI		OR	95% CI		OR	95% CI		OR	95% CI	
Primary payer^a^																		
Not Insured	1.18	1.11	1.26	1.17	1.03	1.34	1.20	0.94	1.52	1.15	0.88	1.50	1.05	0.61	1.81	0.78	0.40	1.51
Private Insurance/Managed Care	Ref			Ref			Ref.			Ref			Ref			Ref		
Medicaid	1.21	1.16	1.26	1.21	1.12	1.32	1.04	0.87	1.26	1.01	0.83	1.23	1.32	0.91	1.90	1.28	0.77	2.12
Medicare	1.06	1.02	1.09	1.08	1.01	1.14	0.95	0.85	1.07	1.03	0.89	1.19	0.96	0.71	1.29	1.00	0.73	1.38
Other Government	1.06	0.96	1.16	1.10	0.93	1.29	1.20	0.57	1.25	0.97	0.62	1.51	0.76	0.48	1.21	1.07	0.38	3.04
State medicaid expansion status (2011–2016)^b^																		
Non‐Expansion States	Ref			Ref			Ref			Ref			Ref.			Ref.		
January 2014 Expansion States	1.03	0.87	1.20	0.86	0.71	1.04	1.03	0.75	1.39	1.21	0.93	1.57	1.17	0.77	1.78	1.54	0.86	2.75
Early Expansion States (2010–2013)	0.68	0.54	0.85	0.64	0.48	0.85	0.60	0.41	0.89	0.34	0.20	0.60	0.46	0.29	0.73	0.44	0.41	1.62
Late Expansion States (after Jan. 2014)	1.02	0.85	1.22	0.77	0.61	0.97	1.33	0.96	1.84	1.04	0.74	1.48	0.83	0.51	1.34	0.72	0.22	2.31
Distance from patient to provider (Crowfly)^c^																		
<2 miles	Ref			Ref			Ref			Ref			Ref.			Ref.		
2–4 miles	0.99	0.93	1.07	0.93	0.86	1.01	0.89	0.77	1.02	1.15	0.92	1.42	1.04	0.63	1.72	1.15	0.72	1.84
5–9 miles	0.98	0.92	1.04	0.95	0.85	1.06	0.93	0.79	1.10	1.10	0.89	1.35	1.13	0.66	1.92	0.76	0.40	1.44
10–19 miles	1.04	0.97	1.11	0.89	0.80	0.99	0.88	0.74	1.05	1.16	0.89	1.52	0.96	0.61	1.52	1.26	0.71	2.23
20–45 miles	1.02	0.95	1.10	0.95	0.80	1.12	1.03	0.84	1.26	1.29	0.73	1.29	1.06	0.66	1.71	1.79	0.99	3.22
>45 miles	0.89	0.80	0.99	0.83	0.69	0.99	0.97	0.75	1.26	1.95	0.98	1.95	1.1	0.71	1.69	1.27	0.77	2.09
Facility type^d^																		
Community Cancer Program	0.88	0.77	1.00	0.89	0.70	1.12	0.82	0.61	1.11	1.18	0.68	2.05	0.78	0.52	1.18	1.28	0.66	2.47
Comprehensive Community Cancer Program	Ref.			Ref			Ref			Ref			Ref			Ref.		
Academic/Research Program	0.96	0.84	1.10	1.07	0.88	1.30	1.14	0.89	1.47	1.31	0.73	2.36	0.75	0.52	1.09	2.34	1.43	3.84
Integrated Network Cancer Program	1.09	0.94	1.27	1.24	1.00	1.52	1.40	1.05	1.84	1.37	1.00	1.88	0.64	0.40	1.04	1.02	0.53	1.97

^a^ Adjusted for age and median household income quartile of patient's zip code.

^b^ Data were restricted to 2011–2016 as the Affordable Care Act started in 2011 (*n* = 445,441). Adjusted for age and census region.

^c^ Adjusted for age, area of residence and census region; Model evaluating Native Hawaiian/Pacific Islander did not include area of residence (urban/rural) due to collinearity.

^d^ Adjusted for age, insurance status, greatest circle distance to care, area of residence, (urban/rural), and census region; Model evaluating Native Hawaiian/Pacific Islander did not include area of residence due to collinearity.

## 4 DISCUSSION

Despite the documented benefits of palliative care toward improved survival and higher patient‐reported quality of life, our study demonstrates that only about one in five patients with aNSCLC utilized palliative care in the US. NH‐Black and Hispanic/Latinx patients with aNSCLC were less likely to utilize palliative care compared to NH‐White patients, which suggests inequitable access to high‐quality cancer care. Inequities observed across racial/ethnic groups in cancer care may stem from structural and societal barriers to equitable access to health care in the US. As such, it is important to characterize the role different aspects of social determinants may play in access to care. Our analyses suggest several health care access measures may influence palliative care use among patients with aNSCLC, including insurance status and the type of cancer treatment facility. Identification of multi‐level determinants of palliative care use introduces the opportunity for intervention development to improve palliative care uptake and deliver high‐quality equitable care.

The benefits of early palliative care intervention among patients with cancer were first demonstrated among patients with metastatic non‐small cell lung cancer in a randomized controlled trial.^13^ The trial demonstrated that patients with aNSCLC who received early palliative care had improved survival (11.6 months vs. 8.9 month), a better quality of life and reported fewer symptoms of depression than patients who received standard of care (i.e. no palliative care). Despite these documented benefits, we found that few patients with aNSCLC used palliative care. Prior research evaluating the prevalence of palliative care use among patients with aNSCLC varies, potentially due to underlying differences in the demographic distribution of the patient population. For example, in a recent study of patients with advanced‐stage lung cancer receiving care at the Veterans Health Administration (i.e. in patients insured by the Veterans Affairs), more than half of patients diagnosed between 2007 to 2013 received palliative care.^16^ Another study leveraging data from Surveillance, Epidemiology, and End Results‐Medicare found that 15% of Medicare‐insured metastatic NSCLC patients received palliative care within the first year after diagnosis, which is similar to our findings.^28^ Given the significant symptom burden experienced by most patients with aNSCLC,^29^ the low use of palliative care in the present study and prior research is of concern. However, we were unable to investigate timeliness of palliative care delivery due to lack of data characterizing date of palliative care provided or sequence of palliative care provision with concurrent curative care. Future work to delineate potential racial/ethnic inequities in time to palliative care receipt among metastatic cancer patients is important and should be prioritized to inform intervention development.

Racial/ethnic inequities exist in palliative care use among patients with aNSCLC, suggesting inequitable delivery of high‐quality cancer care. Our study demonstrates that NH‐Black and Hispanic/Latinx patients with aNSCLC are less likely to use palliative care compared to their NH‐White counterparts. Prior work has focused on racial inequities of palliative care use in the context of end‐of‐life care,^30^, ^31^, ^32^, ^33^ and similar to our work, demonstrated that NH‐Black patients are less likely to receive referral to hospice or to receive palliative care near the end‐of‐life. Several reasons have been attributed to the inequities observed in palliative care use at the end‐of‐life among patients with cancer from minoritized communities, including poor patient‐provider communication regarding the role of palliative care.^34^ Provider training and ability to initiate timely discussions regarding end‐of‐life care and decisions toward fostering positive quality‐of‐life through hospice or complementary supportive care is critical. Although providers support early intervention with palliative care, less than one in five report they would refer aNSCLC patient at diagnosis.^35^ In the context of caring for patients with cancer from minoritized communities, providers are shown to apply implicit bias during clinical‐decision making and may assume patients have limited ability to understand medical decision‐making.^36^ In addition to provider‐level barriers to palliative care use, patient‐level attitudes and perceptions regarding the role of palliative care and treatment near the end‐of‐life can also impact racial/ethnic minority patient's decisions. Recent work has focused on the significance of palliative care use in the context of cultural and religious beliefs among US indigenous populations, such as Native Americans and Alaskan Natives.^37^, ^38^ Limited research exists focused on these populations; however, inequities in palliative care use in the context of end‐of‐life care have been documented among Indigenous populations potentially due to limitations in resources available in Indian Health Services health care facilities.^39^ Our research adds to the limited prior work and shows Indigenous populations are more likely to use palliative care compared to NH‐White patients with aNSCLC. Given that the NCDB data are drawn from CoC‐accredited health care facilities with significant resources, prior barriers to palliative care documented among Indigenous populations may not apply in our research context. Future research focusing on Indigenous US populations should be prioritized, particularly from the patient perspective, to evaluate delivery of palliative care in this context.

When contextualizing the results of these analyses, it is important to consider the types of patients captured in the NCDB. Although the NCDB captures 70% of patients from diverse regions of the country and a large majority of aNSCLC cases in the US, the NCDB is not population‐based. The NCDB only captures patients who received care at a Commission on Cancer (CoC)‐accredited US hospital, which includes over 1500 programs in the US and Puerto Rico. The CoC is a consortium of 56 professional organizations, such as the American Cancer Society, and the Centers for Disease Control and Prevention, which reviews the services and resources available at a specific hospital to ensure they address the full spectrum of the cancer care continuum from prevention to end‐of‐life. As such, CoC‐accredited hospitals are not representative of all clinics in the US, particularly clinics where palliative care may not be made available to patients. However, despite the high‐resource setting of CoC hospitals, it is still important to acknowledge that the data on palliative care services are of uncertain accuracy. Non‐curative surgery, radiation, and systemic therapy as documented by the NCDB may be considered as palliative care resources and do not meet the criteria for early intervention with palliative care. Further, information regarding hospice referral is unavailable and should be evaluated in future studies focused on palliative care use across the care continuum, including end‐of‐life.

In the U.S, lung cancer is one of the most common cancers among adults and the leading cause of cancer‐related death. Racial inequities exist in mortality and outcomes among patients with lung cancer, particularly affecting NH‐Black and Hispanic/Latinx adults. Opportunities exist for improving outcomes after lung cancer diagnosis through palliative care, as demonstrated by the survival benefits when palliative care is introduced early in the cancer treatment trajectory of a patient. However, we continue to observe racial inequities in receipt of palliative care, with only 20% of all adults diagnosed with aNSCLC using palliative care. Future research integrating implementation science principles into intervention development to address palliative care inequities should be prioritized. In particular, interventions informed by research characterizing palliative care use among patients with aNSCLC receiving curative care or those who have declined curative treatment would be of high priority given the potential missed opportunity to treat curable cancers if diagnosed at an early stage. High‐quality and equitable cancer care delivery through equitable use of palliative care among patients with cancer should be prioritized in the US to mitigate the long‐standing effects of racism and historic structural barriers to access to care.

## AUTHOR CONTRIBUTIONS

**Jessica Y Islam:** Conceptualization (lead); formal analysis (equal); methodology (lead); visualization (equal); writing – original draft (lead); writing – review and editing (equal). **Dejana Braithwaite:** Formal analysis (equal); methodology (equal); visualization (equal); writing – review and editing (equal). **Dongyu Zhang:** Formal analysis (equal); methodology (equal); visualization (equal); writing – review and editing (equal). **Yi Guo:** Methodology (equal); writing – review and editing (equal). **Tina Dinesh Tailor:** Conceptualization (equal); methodology (equal); writing – review and editing (equal). **Tomi F Akinyemiju:** Conceptualization (equal); investigation (lead); methodology (equal); resources (lead); supervision (lead); writing – original draft (equal); writing – review and editing (equal).

## FUNDING INFORMATION

This project was conducted without funding support.

## CONFLICTS OF INTEREST

The authors declare no potential conflicts of interest.

## Supporting information


Data S1.

## Data Availability

The data used in this study are available from the National Cancer Database (https://www.facs.org/quality‐programs/cancer/ncdb).
