# Supplementary material for: Racial and ethnic inequities of palliative care use among advanced Non‐Small cell lung cancer patients in the US
Source: Cancer Med. 2022 Dec 19;12(7):8567–80. doi: 10.1002/cam4.5538 (PMC10134338; doi:10.1002/cam4.5538)
Supplement: Supplementary file 1 — Data S1. [file CAM4-12-8567-s001.docx]

**Supplementary Materials**

Figure 1: DAG summarizing minimally sufficient adjustment set to evaluate the relationship between patient’s insurance status and use of palliative care. Patient’s race/ethnicity should not be considered a biological variable but rather a proxy measure of the social experience of racially marginalized communities in the US.


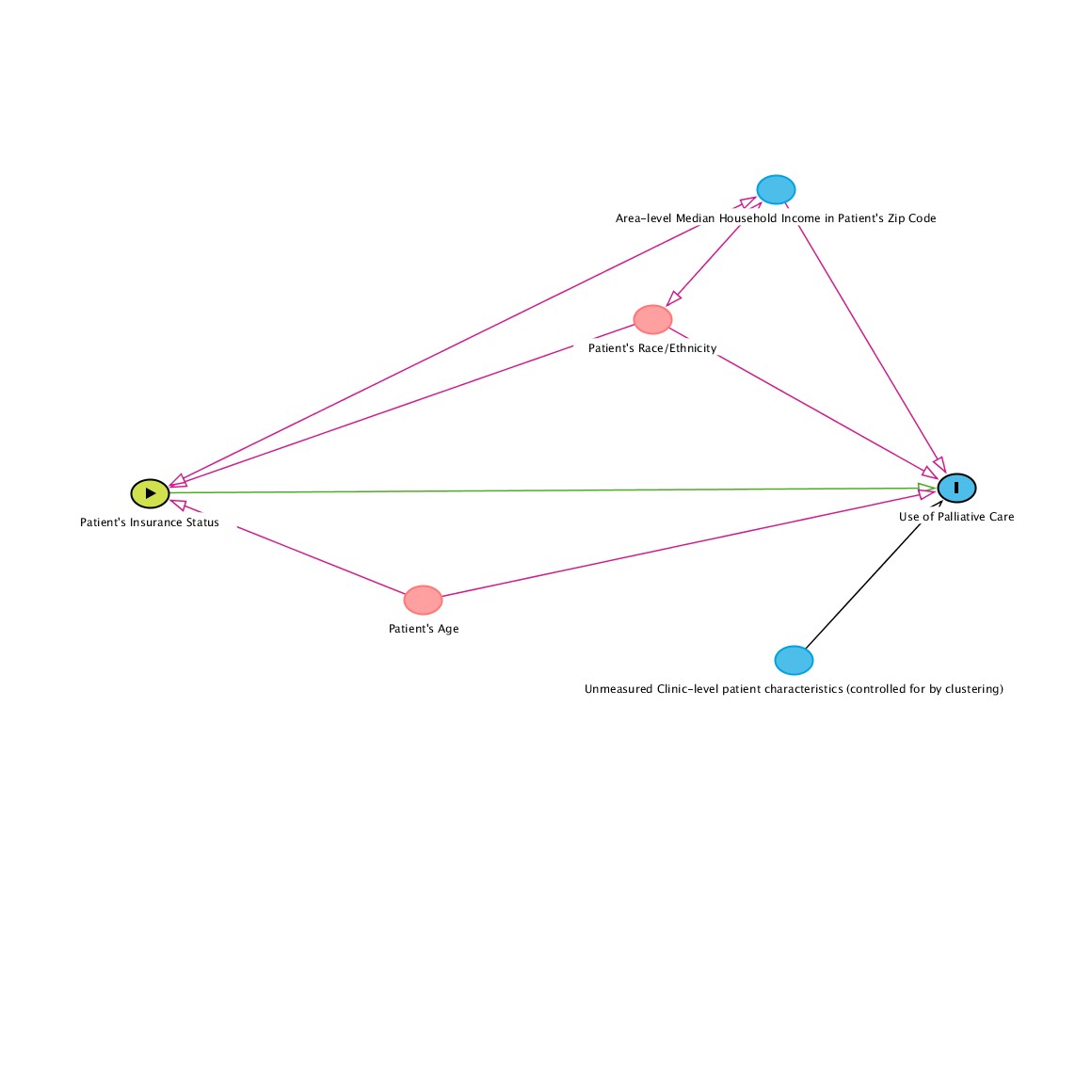


Figure 2: DAG summarizing minimally sufficient adjustment set to evaluate the relationship between the Medicaid Expansion status of the patient’s state of residence with use of palliative care. Patient’s race/ethnicity should not be considered a biological variable but rather a proxy measure of the social experience of racially marginalized communities in the US.


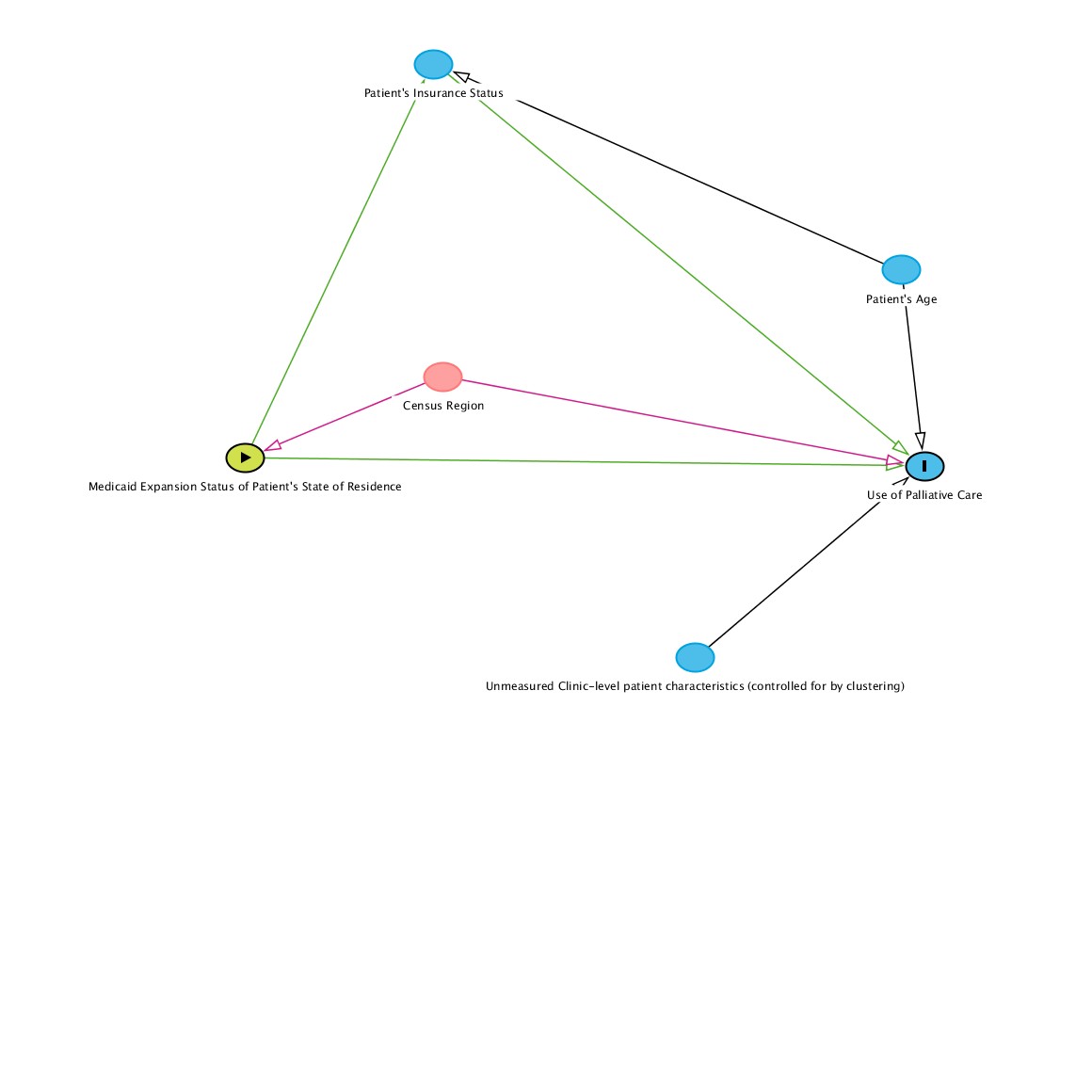


Figure 3: DAG summarizing minimally sufficient adjustment set to evaluate the relationship between the distance to care with use of palliative care. Patient’s race/ethnicity should not be considered a biological variable but rather a proxy measure of the social experience of racially marginalized communities in the US.


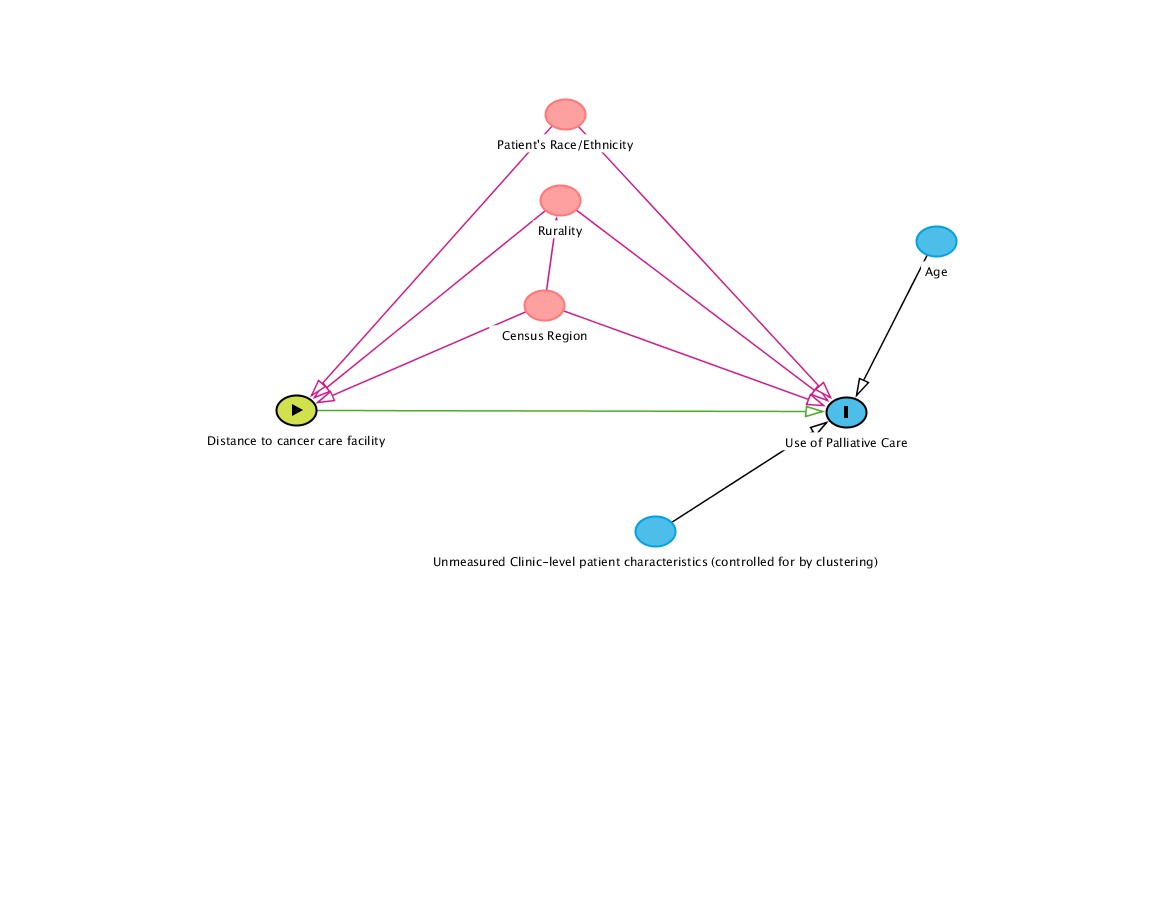


Figure 4: DAG summarizing minimally sufficient adjustment set to evaluate the relationship between the cancer care facility type with use of palliative care. Patient’s race/ethnicity should not be considered a biological variable but rather a proxy measure of the social experience of racially marginalized communities in the US.


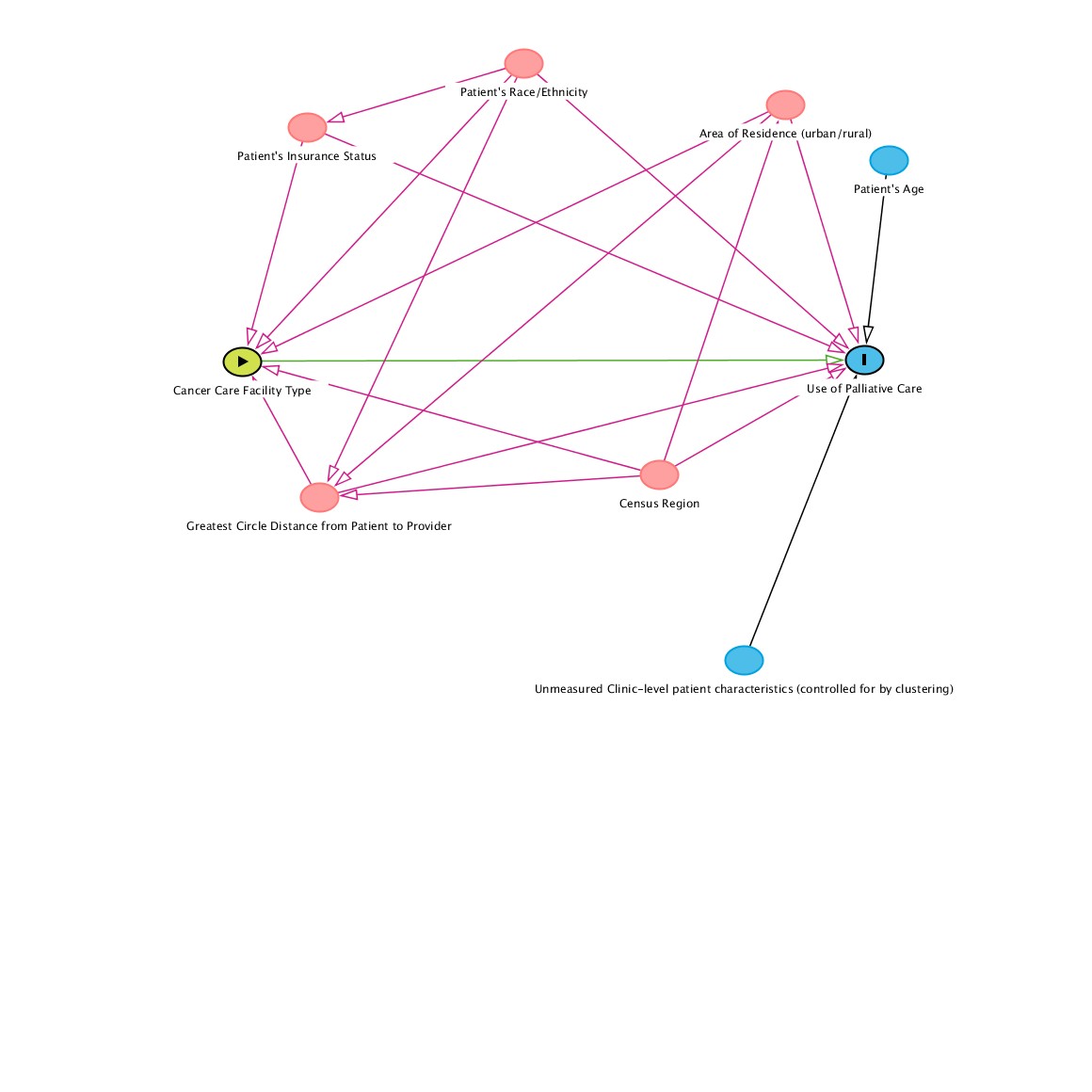


| **Supplementary Table 1: Characteristics among patients with advanced stage (Stage 3/4 or metastatic) non-small cell Lung Cancer with known palliative care receipt status included in Medicaid Expansion analyses (2011-2016) (n = 445,441)** | | | | | | |
| --- | --- | --- | --- | --- | --- | --- |
|  | No Palliative Care  (n = 353,992) | | Received Palliative Care (n = 91,449) | | Total (n = 445,441) | |
|  | No. | Row % | No. | Row % | No. | Col % |
| Age (Mean, SD) | 68.1, 10.9 |  | 67.3, 11.0 |  | 67.9, 10.9 |  |
| Palliative care provided (Col %) |  |  |  |  |  |  |
| No palliative care | 353992 | 100 | 0 | 0 | 353992 | 79.5 |
| Surgery/radiation/chemo only | 0 | 0 | 66138 | 100 | 66138 | 14.8 |
| Pain management only | 0 | 0 | 5893 | 100 | 5893 | 1.3 |
| Combination of surg/rad/chemo and pain management | 0 | 0 | 12727 | 100 | 12727 | 2.9 |
| Type unknown | 0 | 0 | 6691 | 100 | 6691 | 1.5 |
| Sex |  |  |  |  |  |  |
| Male | 191620 | 79.2 | 50313 | 20.8 | 241933 | 54.3 |
| Female | 162372 | 79.8 | 41136 | 20.2 | 203508 | 45.7 |
| Months from Diagnosis to Last Contact or Death |  |  |  |  |  |  |
| >6 months | 125116 | 74.3 | 43226 | 25.7 | 168342 | 37.8 |
| 6-24 months | 112854 | 81.2 | 26066 | 18.8 | 138920 | 31.2 |
| >24 months | 116022 | 84 | 22157 | 16 | 138179 | 31 |
| Race and Ethnicity |  |  |  |  |  |  |
| Non-Hispanic/Latinx White | 275572 | 79.1 | 72601 | 20.9 | 348173 | 78.2 |
| Non-Hispanic/Latinx Black | 42657 | 79.5 | 10970 | 20.5 | 53627 | 12 |
| Hispanic/Latinx | 11776 | 83.4 | 2341 | 16.6 | 14117 | 3.2 |
| Asian | 9859 | 82.5 | 2096 | 17.5 | 11955 | 2.7 |
| American Indian/Alaskan Native | 1044 | 78.3 | 290 | 21.7 | 1334 | 0.3 |
| Native Hawaiian/Pacific Islander | 556 | 73.5 | 200 | 26.5 | 756 | 0.2 |
| Other Race | 11463 | 80.9 | 2704 | 19.1 | 14167 | 3.2 |
| Missing | 1065 | 81.2 | 247 | 18.8 | 1312 | 0.3 |
| Primary Health Insurance Payor |  |  |  |  |  |  |
| Not Insured | 12727 | 76.5 | 3899 | 23.5 | 16626 | 3.7 |
| Private Insurance/Managed Care | 92172 | 79.2 | 24235 | 20.8 | 116407 | 26.1 |
| Medicaid | 27259 | 76.8 | 8232 | 23.2 | 35491 | 8 |
| Medicare | 209956 | 80 | 52444 | 20 | 262400 | 58.9 |
| Other Government | 6081 | 80.4 | 1484 | 19.6 | 7565 | 1.7 |
| Insurance Status Unknown | 5797 | 83.4 | 1155 | 16.6 | 6952 | 1.6 |
| Percent Without a High School Degree Residing in Zip Code (Quartiles) 2012-2016 (Area-Level) |  |  |  |  |  |  |
| >=17.6% | 81397 | 80.9 | 19275 | 19.1 | 100672 | 22.6 |
| 10.9-17.5% | 100386 | 79.5 | 25860 | 20.5 | 126246 | 28.3 |
| 6.3-10.8% | 97595 | 78.9 | 26157 | 21.1 | 123752 | 27.8 |
| <6.3% | 71004 | 78.6 | 19277 | 21.4 | 90281 | 20.3 |
| Missing | 3610 | 80.4 | 880 | 19.6 | 4490 | 1 |
| Median Income of Adults Residing in Zip Code (Quartiles) 2012-2016 (Area-Level) |  |  |  |  |  |  |
| < $40,227 | 77388 | 79.5 | 19974 | 20.5 | 97362 | 21.9 |
| $40,227-50,353 | 84008 | 79.4 | 21837 | 20.6 | 105845 | 23.8 |
| $50,354-63,332 | 82260 | 79.4 | 21354 | 20.6 | 103614 | 23.3 |
| >=$63,333 | 105969 | 79.6 | 27221 | 20.4 | 133190 | 29.9 |
| Missing | 4367 | 80.4 | 1063 | 19.6 | 5430 | 1.2 |
| Rurality |  |  |  |  |  |  |
| Urban | 338054 | 79.5 | 87115 | 20.5 | 425169 | 95.4 |
| Rural | 7617 | 77.8 | 2179 | 22.2 | 9796 | 2.2 |
| Missing | 8321 | 79.4 | 2155 | 20.6 | 10476 | 2.4 |
| State Medicaid Expansion Status (2011-2016)* |  |  |  |  |  |  |
| Non-Expansion States | 138147 | 80.1 | 34397 | 19.9 | 172544 | 38.7 |
| January 2014 Expansion States | 108049 | 77.4 | 31512 | 22.6 | 139561 | 31.3 |
| Early Expansion States (2010-2013) | 56219 | 84.5 | 10275 | 15.5 | 66494 | 14.9 |
| Late Expansion States (after Jan. 2014) | 49477 | 77.1 | 14688 | 22.9 | 64165 | 14.4 |
| Suppressed for Ages 0-39 | 2100 | 78.4 | 577 | 21.6 | 2677 | 0.6 |
| Distance from Patient to Provider (Crowfly) |  |  |  |  |  |  |
| <2 miles | 37566 | 79.4 | 9728 | 20.6 | 47294 | 10.6 |
| 2-4 miles | 71956 | 79.7 | 18365 | 20.3 | 90321 | 20.3 |
| 5-9 miles | 76798 | 79.7 | 19525 | 20.3 | 96323 | 21.6 |
| 10-19 miles | 70190 | 79 | 18701 | 21 | 88891 | 20 |
| 20-45 miles | 59490 | 78.8 | 16005 | 21.2 | 75495 | 16.9 |
| >45 miles | 37992 | 80.6 | 9125 | 19.4 | 47117 | 10.6 |
| Charlson-Deyo Score |  |  |  |  |  |  |
| 0 | 210972 | 79.6 | 54084 | 20.4 | 265056 | 59.5 |
| 1 | 92443 | 79.3 | 24123 | 20.7 | 116566 | 26.2 |
| 2 | 34124 | 79.5 | 8814 | 20.5 | 42938 | 9.6 |
| >=3 | 16453 | 78.8 | 4428 | 21.2 | 20881 | 4.7 |
| Treatment Facility Type |  |  |  |  |  |  |
| Community Cancer Program | 40943 | 81.2 | 9476 | 18.8 | 50419 | 11.3 |
| Comprehensive Community Cancer Program | 153651 | 79.8 | 38999 | 20.2 | 192650 | 43.2 |
| Academic/Research Program | 109646 | 79.1 | 28938 | 20.9 | 138584 | 31.1 |
| Integrated Network Cancer Program | 47652 | 78 | 13459 | 22 | 61111 | 13.7 |
| Missing | 2100 | 78.4 | 577 | 21.6 | 2677 | 0.6 |
| Census region |  |  |  |  |  |  |
| Northeast | 69770 | 76 | 22066 | 24 | 91836 | 20.6 |
| South | 140421 | 80.8 | 33434 | 19.2 | 173855 | 39 |
| Midwest | 92020 | 77.6 | 26566 | 22.4 | 118586 | 26.6 |
| West | 49681 | 84.9 | 8806 | 15.1 | 58487 | 13.1 |
| Missing | 2100 | 78.4 | 577 | 21.6 | 2677 | 0.6 |
| Year of Diagnosis |  |  |  |  |  |  |
| 2011 | 57495 | 80.8 | 13636 | 19.2 | 71131 | 16 |
| 2012 | 58442 | 80.2 | 14391 | 19.8 | 72833 | 16.4 |
| 2013 | 59050 | 79.1 | 15561 | 20.9 | 74611 | 16.7 |
| 2014 | 60047 | 78.7 | 16217 | 21.3 | 76264 | 17.1 |
| 2015 | 60682 | 79 | 16125 | 21 | 76807 | 17.2 |
| 2016 | 58276 | 79 | 15519 | 21 | 73795 | 16.6 |
| Grade |  |  |  |  |  |  |
| Well differentiated, differentiated, NOS | 10186 | 87.3 | 1480 | 12.7 | 11666 | 2.6 |
| Moderately differentiated, moderately well differentiated, intermediate differentiation | 49151 | 85.1 | 8621 | 14.9 | 57772 | 13 |
| Poorly differentiated | 99270 | 80.7 | 23666 | 19.3 | 122936 | 27.6 |
| Undifferentiated, anaplastic | 3926 | 79.7 | 997 | 20.3 | 4923 | 1.1 |
| Cell type not determined, not stated or not applicable, unknown primaries, high grade dysplasia | 191459 | 77.2 | 56685 | 22.8 | 248144 | 55.7 |
| NCDB Analytic Stage Group |  |  |  |  |  |  |
| Stage III | 136012 | 94 | 8656 | 6 | 144668 | 32.5 |
| Stage IV | 217980 | 72.5 | 82793 | 27.5 | 300773 | 67.5 |
| *Data were restricted to 2011-2016 as the Affordable Care Act started in 2011 (n=445,441). | | | | | | |
